# Robotic Surgery in the Treatment of Combined Wilkie’s and Dunbar’s Syndromes: A Case Report

**DOI:** 10.3390/life16030425

**Published:** 2026-03-05

**Authors:** Vladimir A. Porhanov, Roman A. Vinogradov, Aslan B. Zakeryaev, Khabib A. Kurbanov, Tarlan E. Bakhishev, Marina R. Pchegatluk, Alim M. Namitokov, Amirlan A. Sozaev, Anastasia V. Erastova

**Affiliations:** 1 Scientific Research Institute–Ochapovsky Regional Clinical Hospital #1, Krasnodar, 167, 1st May Str., 350086 Krasnodar, Russia; vladimirporhanov@mail.ru (V.A.P.); viromal@mail.ru (R.A.V.); aslan.zakeryaev@gmail.com (A.B.Z.); tarlan.bakhishev@yandex.ru (T.E.B.); 2 Kuban State Medical University, Krasnodar, 4, Sedina Street, 350086 Krasnodar, Russia; khabibkurbanovv@mail.ru (K.A.K.); pchegatlukmarina@yandex.ru (M.R.P.); sozaev.amirlan@mail.ru (A.A.S.); nastya.erastowa@gmail.com (A.V.E.)

**Keywords:** Wilkie’s syndrome, Dunbar’s syndrome, Superior Mesenteric Artery Syndrome, Celiac Artery Compression Syndrome, robot-assisted surgery, da Vinci robot, vascular surgery, minimally invasive technologies, vascular compression syndromes

## Abstract

In clinical practice, the coexistence of Superior Mesenteric Artery (SMA) syndrome (also known as Wilkie’s syndrome) and Celiac Artery Compression Syndrome (also referred to as Dunbar’s syndrome) is extremely rare. This combined pathology is characterized by simultaneous impairment of blood flow in the celiac trunk and compression of the duodenum, which complicates both diagnosis and treatment strategy selection. Traditional open surgical correction is associated with significant invasiveness due to the complexity of the anatomical relationships involved. Minimally invasive approaches, including robot-assisted surgery, allow precise dissection within confined anatomical spaces. This article presents a clinical case of simultaneous robot-assisted decompression of the celiac trunk and duodenum using the da Vinci Xi system. The case demonstrates the technical feasibility of a combined minimally invasive approach for the management of concurrent vascular and duodenal compression.

## 1. Introduction

Superior Mesenteric Artery (SMA) syndrome, also known as Wilkie’s syndrome, and Celiac Artery Compression Syndrome, also referred to as Dunbar’s syndrome, are rare vascular compression disorders caused by external compression of visceral structures by adjacent anatomical elements [1,2,3]. SMA syndrome involves compression of the horizontal (third) portion of the duodenum between the superior mesenteric artery and the abdominal aorta. In contrast, Celiac Artery Compression Syndrome results from extrinsic compression of the celiac trunk by the median arcuate ligament of the diaphragm, which may lead to symptoms of chronic mesenteric ischemia [1,2,4,5].

Both syndromes predominantly affect young and middle-aged women and are frequently associated; according to the literature, combined compression of the duodenum and celiac trunk occurs in approximately 18.9% of patients with Wilkie’s syndrome [2]. Despite low prevalence (0.013–0.3% for Wilkie’s syndrome and about 2 per 100,000 for Dunbar’s syndrome), these conditions hold significant clinical importance due to chronic pain, digestive disorders, and persistent reduction in patients’ quality of life [1,4].

Traditional surgical treatment for Wilkie’s and Dunbar’s syndromes aims to eliminate vascular compression and restore digestive tract patency or blood flow. Various methods are employed: duodenojejunostomy, gastrojejunostomy, division of the ligament of Treitz (Strong’s procedure) for Wilkie’s syndrome, as well as division of the median arcuate ligament and neurolysis of the celiac plexus for Dunbar’s syndrome [5,6,7,8,9,10,11,12,13]. However, all these interventions, especially when performed via traditional open approaches, are characterized by high invasiveness, significant blood loss, and risk of complications (intestinal obstruction, anastomotic leakage, dumping syndrome, thrombosis, bowel ischemia), reaching 6–15% in the literature, with mortality up to 5% [14].

With the advancement of minimally invasive surgery, laparoscopic techniques have been increasingly adopted for abdominal vascular compression syndromes. In Superior Mesenteric Artery (SMA) syndrome, laparoscopic duodenojejunostomy demonstrated symptom improvement in 92% of patients in a small retrospective series, with no reported mortality and short hospitalization [6]. However, intracorporeal reconstruction in a confined space near the aorta and mesenteric vessels remains technically demanding and inherently carries risks of leakage, stricture, and postoperative obstruction. Similarly, laparoscopic division of the median arcuate ligament for Celiac Artery Compression Syndrome (Dunbar’s syndrome) has been shown to be feasible and safe [15]. Nevertheless, precise vascular dissection around the celiac trunk and adjacent neural structures may be limited by restricted instrument mobility, tremor transmission, and suboptimal ergonomics. Although laparoscopy represents a major advance over open surgery, its technical constraints may affect surgical precision in complex vascular decompression procedures. Robot-assisted systems provide three-dimensional visualization, tremor filtration, and articulated instruments, potentially enabling more accurate and controlled vascular dissection, particularly in combined compression syndromes. The aim of this report is to present a rare case of combined SMA syndrome and Celiac Artery Compression Syndrome treated with simultaneous robot-assisted decompression and to highlight the technical aspects of this minimally invasive approach.

## 2. Materials and Methods

### 2.1. Case Presentation

A 37-year-old male patient was admitted to the surgical department of the Ochapovsky Regional Clinical Hospital with complaints of abdominal distension, difficulty passing food, nausea, shortness of breath, and general weakness. The patient reported a six-year history of progressive symptoms, with marked deterioration since February 2025. Conservative therapy, including regular use of osmotic laxatives (macrogol), provided only temporary relief and became ineffective by May 2025, which prompted hospitalization.

**Figure 1:**
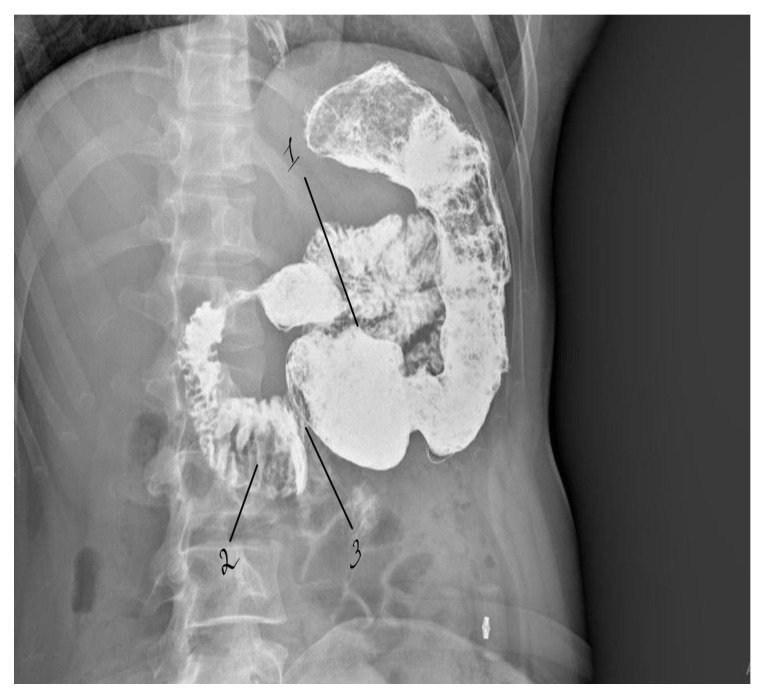
Preoperative contrast study demonstrating dilation of the proximal duodenum with delayed passage of contrast at the level of aortomesenteric compression. 1—stomach; 2—dilated duodenum proximal to the compression site; 3—area of aortomesenteric compression.

Triplex duplex ultrasound examination of the visceral arteries demonstrated hemodynamically significant stenosis of the celiac trunk, with a peak systolic velocity (PSV) exceeding 300 cm/s, consistent with severe (>70%) arterial compression. Contrast-enhanced abdominal radiography demonstrated delayed passage of contrast through the inferior horizontal (third) portion of the duodenum (Figure 1).

**Figure 2:**
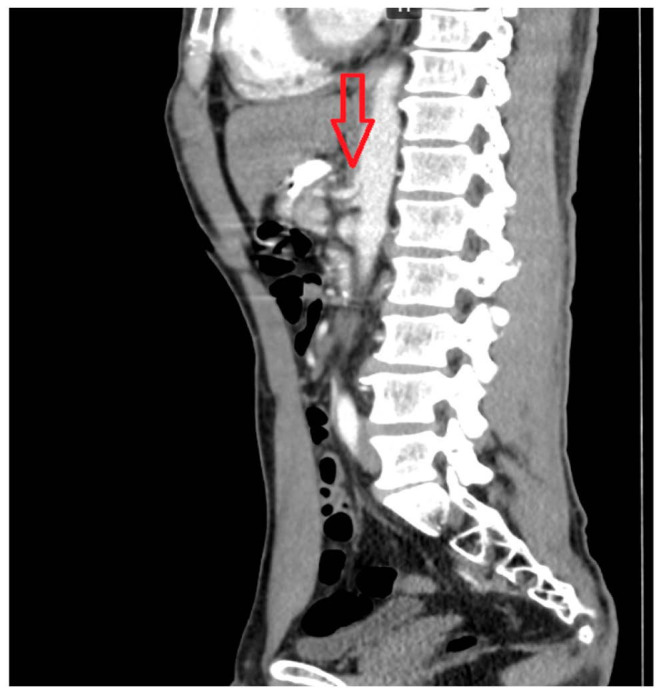
Contrast-enhanced multislice computed tomography (MSCT) of the abdominal organs demonstrating extravascular compression of the celiac trunk by the median arcuate ligament of the diaphragm (arrow).

**Figure 3:**
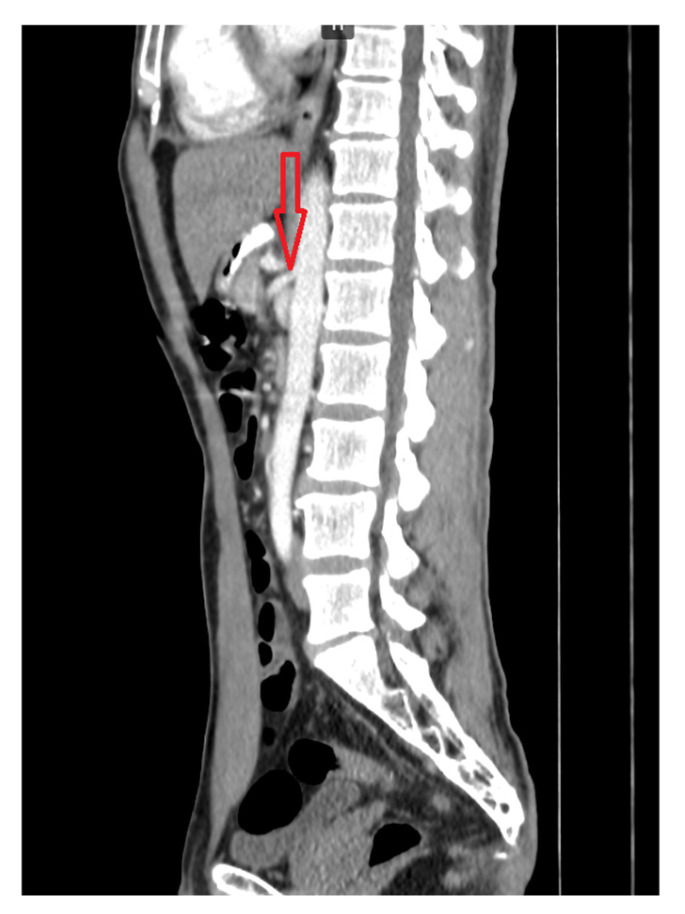
Contrast-enhanced multislice computed tomography (MSCT) showing compression of the horizontal (third) part of the duodenum between the superior mesenteric artery and the abdominal aorta (arrow).

Multislice computed tomography (MSCT) of the abdomen and pelvis with contrast enhancement confirmed extravascular compression of the celiac trunk by the median arcuate ligament of the diaphragm, as well as compression of the horizontal part of the duodenum between the superior mesenteric artery and the abdominal aorta (Figure 2 and Figure 3). The proximal segment of the celiac trunk demonstrated approximately 80% focal luminal narrowing at its origin caused by extrinsic compression from the median arcuate ligament.

Based on the combined clinical and instrumental findings, the diagnosis was established as compression stenosis of the horizontal portion of the duodenum (Wilkie’s syndrome), complicated by gastrostasis and gastroptosis, in combination with celiac trunk compression (Dunbar’s syndrome). Given the failure of conservative treatment, surgical intervention was indicated.

### 2.2. Surgical Technique

The procedure was performed under general endotracheal anesthesia using the da Vinci^®^ Xi robotic system (Intuitive Surgical, Sunnyvale, CA, USA). After induction of pneumoperitoneum with carbon dioxide via a Veress needle placed 2 cm above the umbilicus, an 8 mm optical trocar was inserted. Additional robotic trocars were positioned under direct visualization, including ports in the left epigastric, suprapubic, and left iliac regions, as well as a 12 mm assistant trocar. The robotic system was docked in the upper abdominal configuration.

**Figure 4:**
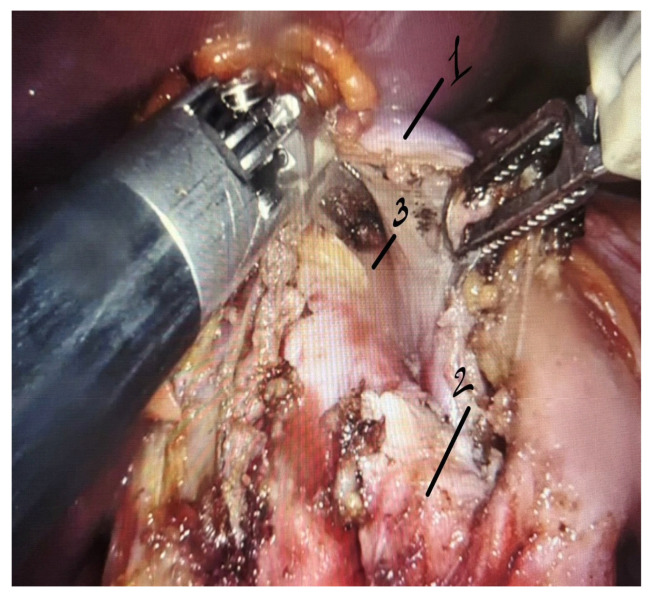
Intraoperative robotic view during dissection of the median arcuate ligament of the diaphragm with exposure and decompression of the celiac trunk. 1—median arcuate ligament; 2—celiac trunk; 3—abdominal aorta.

The left lobe of the liver was retracted cranially. The peritoneum overlying the celiac trunk was incised, and the common hepatic, left gastric, and splenic arteries were sequentially isolated and controlled with soft vessel loops. Fibrous bands of the median arcuate ligament and fibers of the celiac plexus compressing the celiac trunk were carefully divided using robotic instruments. Upon completion of decompression, restoration of pulsatile blood flow along the entire length of the celiac trunk was visually confirmed (Figure 4).

**Figure 5:**
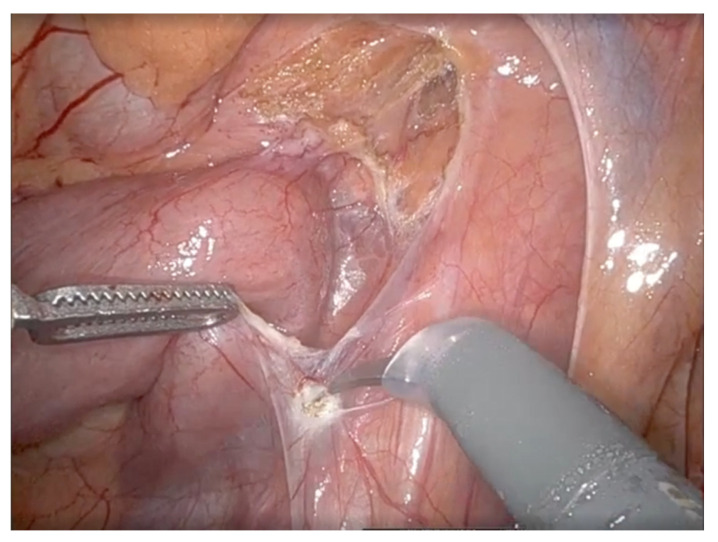
Intraoperative robotic view showing incision of the posterior peritoneal layer along the lateral border of the horizontal part of the duodenum during duodenal mobilization.

**Figure 6:**
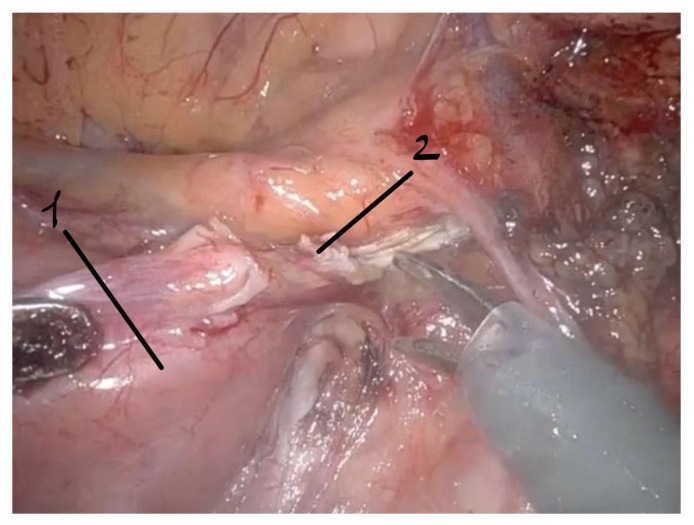
Intraoperative robotic view of division of the ligament of Treitz enabling mobilization of the duodenum from the aortomesenteric compression zone. 1—duodenum; 2—ligament of Treitz.

Subsequently, duodenal decompression was performed. The posterior peritoneal layer was incised along the lateral border of the horizontal part of the duodenum, followed by stepwise mobilization of the intestine (Figure 5). Mobilization was continued cranially with division of the ligament of Treitz, which allowed complete release of the duodenum from the aortomesenteric compression zone (Figure 6). After decompression, the duodenum demonstrated adequate mobility with no visual signs of ischemia.

**Figure 7:**
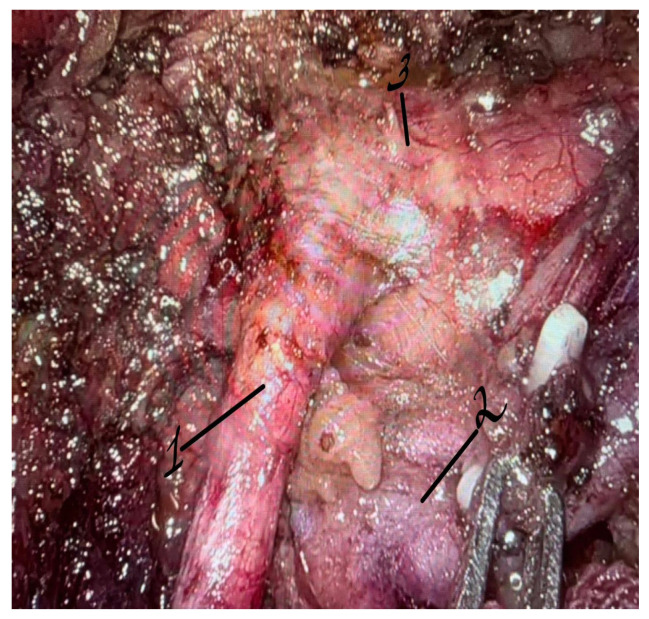
Robotic exposure and skeletonization of the proximal superior mesenteric artery after division of connective tissue attachments, confirming complete vascular decompression. 1—superior mesenteric artery; 2—left renal vein; 3—abdominal aorta.

Additionally, skeletonization of the proximal superior mesenteric artery was performed by dividing connective tissue bands fixing the vessel to the anterior surface of the aorta (Figure 7). Hemostasis was complete at the end of the procedure.

## 3. Results

**Figure 8:**
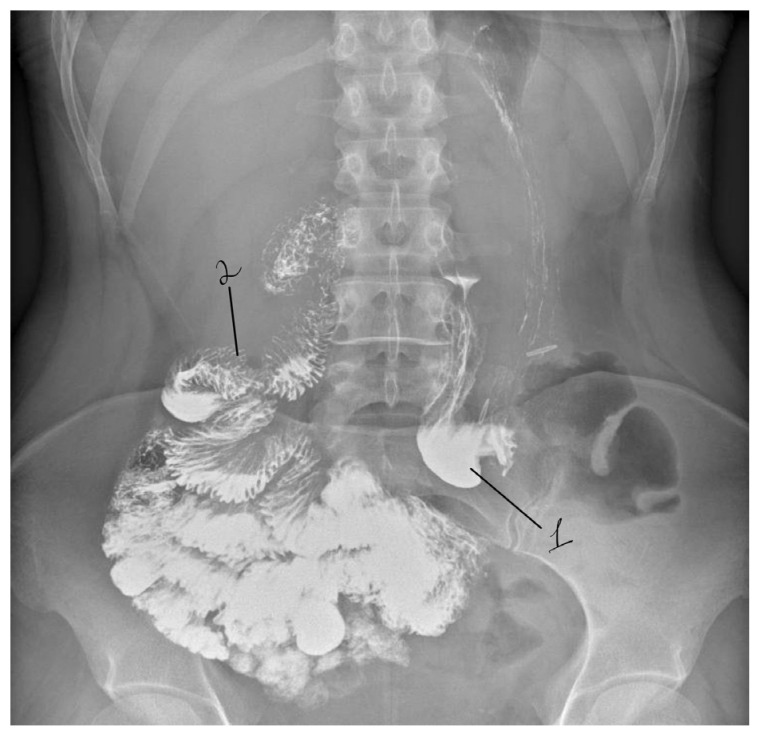
Postoperative contrast study demonstrating restored duodenal passage with free flow of contrast through the stomach and duodenum, with no evidence of residual obstruction. 1—stomach; 2—inferiorly repositioned duodenum following decompression.

The total operative time was 125 min, and estimated blood loss was approximately 100 mL. No intraoperative complications occurred. The patient was extubated in the operating room and transferred to the surgical ward in stable condition. On postoperative day two, the safety drain was removed, and independent oral intake was resumed. The postoperative course was uneventful, and the patient was discharged on postoperative day five in satisfactory condition with complete resolution of obstructive gastrointestinal symptoms (Figure 8). Control duplex ultrasound performed on postoperative day 4 demonstrated a reduction in peak systolic velocity in the celiac trunk to 180 cm/s, indicating resolution of hemodynamically significant stenosis.

**Figure 9:**
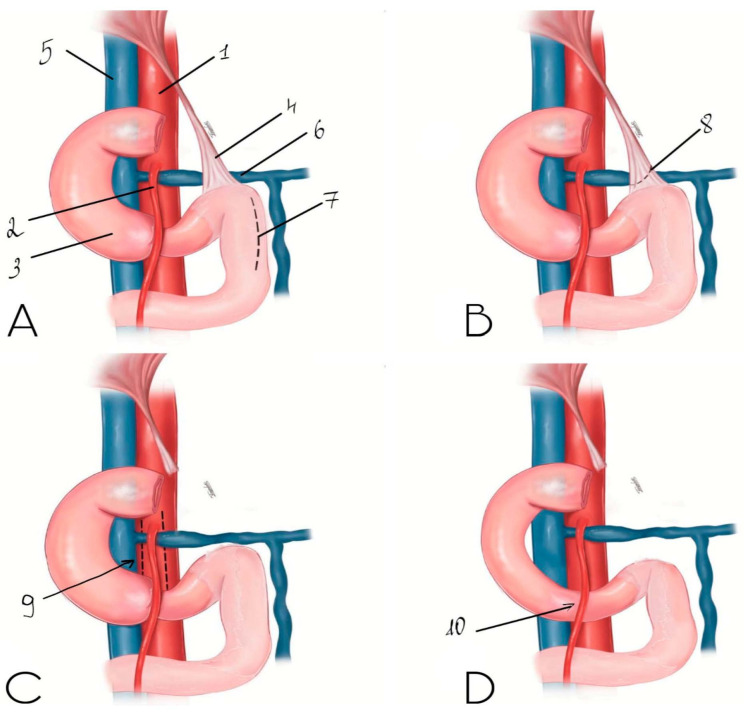
Schematic representation of the final stage of duodenal and vascular decompression after robotic-assisted surgery. (**A**) Dissection of the posterior peritoneal layer. (**B**) Division of the ligament of Treitz. (**C**) Division of connective tissue bands at the origin of the superior mesenteric artery. (**D**) Final anatomical configuration after complete decompression. 1—abdominal aorta; 2—superior mesenteric artery; 3—horizontal part of the duodenum; 4—ligament of Treitz; 5—inferior vena cava; 6—left renal vein; 7—area of dissected posterior peritoneal layer; 8—area of dissected ligament of Treitz; 9—schematic representation of connective tissue band dissection near the origin of the superior mesenteric artery; 10—schematic representation of the final decompression.

The final anatomical configuration achieved after simultaneous vascular and duodenal decompression is schematically illustrated in Figure 9, demonstrating restoration of physiological relationships between the duodenum, superior mesenteric artery, and abdominal aorta.

At 2-month follow-up, the patient remained asymptomatic, reporting complete resolution of postprandial pain, nausea, and obstructive symptoms. Body weight increased by 3 kg compared to the preoperative value. Repeated duplex ultrasound confirmed stable celiac trunk hemodynamics with peak systolic velocity remaining 180 cm/s. No signs of recurrent duodenal obstruction were observed clinically.

## 4. Discussion

Vascular compression syndromes are polyetiological diseases based on disrupted anatomical–topographical relationships between visceral vessels and surrounding structures [1,2,3]. Despite differences in anatomical location, both syndromes share a compressive nature: in Dunbar’s syndrome, extravascular compression of the celiac trunk occurs, while in Wilkie’s syndrome, the duodenum is compressed between the SMA and the aorta.

In Wilkie’s syndrome, duodenal compression results from a narrowing of the aortomesenteric angle due to reduced perivascular adipose tissue or anatomical anomalies of intestinal fixation [1,3]. This leads to impaired content passage, gastrostasis formation, and secondary dilation of the proximal gastrointestinal tract; chronic obstruction is accompanied by inflammatory–dystrophic changes in the mucosa and delayed emptying, perpetuating a vicious cycle of decompensation.

Dunbar’s syndrome is based on compression of the celiac trunk by the fibrous median arcuate ligament of the diaphragm, followed by stenosis of the artery’s origin and the development of abdominal ischemia [4,5]. When the celiac plexus is involved, pronounced autonomic-pain manifestations occur. The combination of Dunbar’s and Wilkie’s syndromes occurs in approximately 18–20% of patients with SMA syndrome and significantly worsens the clinical picture: severe postprandial pain, nausea, bloating, early satiety, and progressive weight loss [2,5].

Diagnosis relies on a combination of clinical signs and instrumental methods. CT angiography with 3D reconstruction provides reliable anatomical assessment (aortomesenteric angle, distance between the aorta and SMA, degree of celiac trunk compression), Doppler ultrasound offers quantitative hemodynamic assessment (peak flow velocity >200 cm/s in the celiac trunk indicates significant compression), and contrast-enhanced abdominal radiography documents delayed contrast passage through the duodenum, confirming Wilkie’s syndrome [5,12,16].

Therapeutic management for Wilkie’s syndrome typically begins with conservative measures: gastric decompression, enteral/parenteral nutrition, and weight correction to restore perivascular adipose tissue and normalize the aortomesenteric angle [1,6]. If conservative therapy is ineffective, surgical correction is indicated. Classic operative methods–gastrojejunostomy, duodenojejunostomy, division of the ligament of Treitz (Strong’s procedure), and, in select cases, SMA transposition–demonstrate consistent effectiveness but are associated with risks of postoperative complications (6–15%) and mortality up to 5% [6,7,8,9,10,14].

Surgical correction of Dunbar’s syndrome (division of the median arcuate ligament, neurolysis of the celiac plexus) remains the standard for hemodynamically significant compression. However, open approaches are characterized by high invasiveness and risks of vascular and neural complications. Endovascular methods (balloon angioplasty, stenting) do not address the extravascular compressive factor and thus have a limited role in primary compression [12,13].

Minimally invasive technologies, particularly laparoscopic and robot-assisted surgery, have shown advantages in decompressing vascular compression syndromes: enhanced visualization, precision dissection within confined spaces, and more delicate manipulation of vessels and intestine reduce intraoperative trauma and may decrease the frequency of early postoperative complications [8,13,17,18,19].

Combined robot-assisted decompression of the celiac trunk with duodenal mobilization (incision of the posterior peritoneal layer along the lateral border and division of the ligament of Treitz) represents a pathogenetically sound approach for combined compression. It simultaneously addresses vascular and intestinal obstruction, restoring physiological blood flow and content passage with minimal invasiveness. Our clinical experience confirms that robot-assisted technique is a logical and promising extension of modern surgical approaches for combined vascular compression syndromes [5,8,12,13].

However, as this report describes a single clinical case, conclusions regarding the generalizability, safety, and effectiveness of simultaneous robot-assisted decompression should be made with caution. Further studies with larger cohorts are necessary to confirm these findings.

## 5. Conclusions

The combination of SMA syndrome and Celiac Artery Compression Syndrome represents a rare and diagnostically challenging condition requiring comprehensive evaluation. This case demonstrates that simultaneous robot-assisted decompression is technically feasible and allows minimally invasive correction of both vascular and duodenal compression. Further studies with larger patient cohorts and long-term follow-up are required to assess the reproducibility and long-term outcomes of this approach.

## Data Availability

Data supporting the findings of this study are available from the corresponding author upon reasonable request.

## Abbreviations

The following abbreviations are used in this manuscript:

SMA	Superior Mesenteric Artery
MSCT	Multislice Computed Tomography
IRB	Institutional Review Board
CT	Computed Tomography
MAL	Median Arcuate Ligament
